# Combined Fluorescence-Guided Surgery with 5-Aminolevulinic Acid and Fluorescein in Glioblastoma: Technical Description and Report of 100 Cases

**DOI:** 10.3390/cancers16162771

**Published:** 2024-08-06

**Authors:** Alessandro Pesaresi, Pietro La Cava, Marta Bonada, Pietro Zeppa, Antonio Melcarne, Fabio Cofano, Pietro Fiaschi, Diego Garbossa, Andrea Bianconi

**Affiliations:** 1 Neurosurgery Unit, Department of Neuroscience, University of Turin, Via Cherasco 15, 10126 Turin, Italyanmelcarne@gmail.com (A.M.); fabio.cofano@unito.it (F.C.);; 2 Department of Neurosurgery, Fondazione IRCCS Istituto Neurologico Carlo Besta, Via Celoria 11, 20133 Milan, Italy; 3 Division of Neurosurgery, Ospedale Policlinico San Martino, IRCCS for Oncology and Neurosciences, Largo Rosanna Benzi 10, 16132 Genoa, Italy; 4 Department of Neuroscience, Rehabilitation, Ophthalmology, Genetics and Maternal and Child Health, University of Genoa, Largo Rosanna Benzi 10, 16132 Genoa, Italy

**Keywords:** glioma, glioblastoma, fluorescence, aminolevulinic acid, fluorescein, maximal safe resection

## Abstract

**Simple Summary:**

This study explores the use of fluorescence-guided resection in glioblastoma surgery, focusing on the combined use of 5-aminolevulinic acid and sodium fluorescein. By analyzing 100 cases from our medical center, we aimed to address concerns about fluorescence-guided resection and share our findings. The dual use of 5-aminolevulinic acid and fluorescein enhances the extent of tumor resection and reduces false positives without increasing adverse effects. In our experience, fluorescein guided the initial resection phase, while 5-aminolevulinic acid identified tumor spots within the surgical cavity, achieving gross total resection in 96% of cases and supra-maximal resection in 11%. This combined approach appears promising for improving outcomes in glioblastoma patients.

**Abstract:**

Background: Fluorescence-guided resection (FGR) of glioblastomas has been previously explored with the use of 5-amivelulinic acid (5-ALA) and sodium fluoresceine (SF), allowing us to maximize the extent of resection (EoR). In this study, we highlight the most relevant concerns regarding this technique and present the methods and results from the experience of our center. Methods: A case series of 100 patients operated on in AOU Città della Salute e della Scienza in Turin with a histological diagnosis of glioblastoma (grade IV, according to WHO 2021) was retrospectively analyzed. Both 5-ALA and SF were administered and intraoperatively assessed with an optical microscope. Results: 5-ALA is the only approved drug for FGR in glioblastoma, reporting an increased EoR. Nevertheless, SF can be positively used in addition to 5-ALA to reduce the risk of false positives without increasing the rate of adverse effects. In our experience, SF was used to guide the initial phase of resection while 5-ALA was used to visualize tumor spots within the surgical cavity. In 96% of cases, gross total resection was achieved, with supra-maximal resection in 11% of cases. Conclusions: Combined FGR using 5-ALA and SF seems to be a promising method of increasing the extent of resection and to improving the prognosis in glioblastoma patients.

## 1. Introduction

Glioblastomas (GBMs) are the most frequent primary brain tumors and still present a poor prognosis [1,2]. Despite recent technological advancements in GBM treatment [3], surgery remains the first fundamental step in the multimodal treatment of these tumors, aiming to improve not only overall survival (OS) but also quality of life (QoL) [4,5]. Previous studies have analyzed the impact of several factors on the OS of patients affected by GBM [6,7,8], but gross total resection (GTR) remains the most relevant. Nevertheless, even if extent of resection (EoR) > 98% is an essential prognostic factor to consider, the prognosis of GBM remains poor due to the infiltrative nature of the disease [9]. Considerable efforts have been devoted to achieving maximal safe resection, both preoperatively and intraoperatively [10,11,12,13,14,15]. In this context, the main surgical focus concerns the resection of tumor borders going beyond the area of contrast enhancement. In fact, tumor cells infiltrate the region around the enhancing area without damaging the blood–brain barrier (BBB), so that the specificity of gadolinium is reduced in this area, possibly hindering the achievement of a real GTR [16]. An exact intraoperative differentiation of neoplastic tissue from normal parenchyma can be challenging, only relying on visual and tactile information. In this context, fluorescence-guided resection (FGR) has proven to be a useful tool to address these issues. So far, two main compounds have been extensively used in malignant glioma surgery: 5-amivelulinic acid (5-ALA) and sodium fluoresceine (SF). However, the two fluorescent dyes differ greatly from each other, with consequent pros and cons to their use. Up to date, many studies have compared the usefulness of 5-ALA and SF in terms of GTR, even if recent studies showed that using 5-ALA and SF simultaneously could improve both GTR and OS in comparison with the administration of one dye alone [17,18,19]. The objective of this study is to propose and describe a surgical technique that utilizes both fluorophores sequentially, aiming to maximize their advantages and minimize their disadvantages. To support this technique, we present a case series of 100 patients affected by GBM that have undergone surgical resection with the double guidance of both 5-ALA and SF. 

## 2. Description of the Surgical Technique and Fluorophores’ Characteristics 

### 2.1. Mechanism of Action, Administration, Intraoperative Use, and Adverse Effects

SF accumulates in neoplastic regions because of passive diffusion through the sites of BBB breakdown [20]. Sharing the same mechanism of action of gadolinium, the distribution of SF in tumor tissue roughly overlaps with the contrast-enhanced area on magnetic resonance imaging (MRI), while 5-ALA, being a metabolic tracer, is not limited by the disruption of the BBB and highlights the infiltrative part of the tumor beyond the enhancing nodule in the FLAIR hyperintense area. However, the diffusion of SF may sometimes exceed the contrast-enhancing region, probably due to the lower molecular weight of SF compared to gadolinium [21]. SF administration occurs intravenously at dosage between 3 and 5 mg/kg at the time of anesthesia induction [22], even if recent studies support the use of low-dose SF (1–4 mg/kg) [23] or ultra-low-dose SF (0.5–1 mg/kg) [24] for glioma surgery. It is completely washed out within 24 h (Table 1).

Briefly, 5-ALA is characterized by a metabolic accumulation within the epithelial and tumoral tissues, including GBMs, which are particularly able to accumulate protoporphyrin IX (PPIX) [25]. This is mainly attributable to two alterations generally present in cancer cells: the reduced activity of the ferrochelatase enzyme, which converts PPIX into heme, and the decreased concentration of the ATP-binding Cassette Subfamily B Member 2 (ABCG2) transporter, which leads to a reduced efflux of PPIX. Both these factors, associated with a general increase in the heme biosynthesis pathway activity, result in an accumulation of PPIX within tumor cells, including glioma cells [26]. Moreover, 5-ALA is orally administrated at a dosage of 20 mg/kg 3 h before induction of anesthesia, and the peak of fluorescence can be expected after about 6–8 h, with fluorescence visibility being noticeable around 3 h after administration [27]. The small size of the molecule allows 5-ALA to be rapidly absorbed from the intestine and cleared from plasma within 2 h of administration [26] (Table 1).

SF is excited by a light wavelength ranging from 460 to 500 nm and emits fluorescent radiation in the wavelength ranging from 540 to 690 nm [28], while 5-ALA requires a filtered xenon light to give blue-violet light with a wavelength from 370 to 440 nm and an emission filter, allowing visualization of red fluorescence, which has a peak at 635 and 704 nm [27]. This implies that each compound requires a modified microscope with a dedicated light source for excitation and an emission filter for optimal fluorescence visualization. This technical issue has been addressed by modern microscopes, which allow the alternative use of both BLUE 400 and YELLOW 560 to detect 5-ALA and SF, respectively [17].

Intraoperative visualization of 5-ALA allows us to further distinguish different patterns: the necrotic area is usually marked by absent/inhomogeneous pink-red fluorescence, solid tumors present bright fluorescence intensity, and the transitional area of infiltrating and invasive brain tissue shows faint fluorescence [29]. On the other hand, different fluorescence patterns using SF have never been reported or classified, even if non-neoplastic structures can be fluorescent, because of tissue manipulation, edema bulk flow, dose, and timing administration [30].

Both the dyes are well tolerated, presenting low rates of adverse events: 5-ALA is mostly associated with minimal liver function alteration, temporary hypotension, and photosensitivity for the first 24 h after application [31], thus requiring the patient to avoid direct light exposure before and after surgery. Adverse events related to administration of SF have been widely evaluated due to its widespread use in angiographic procedures; these include nausea, vomiting and flushing/itching/hives, and they are usually dose-related [32].

### 2.2. Surgical Technique

All patients were treated with the aim of achieving a maximal safe resection of the contrast-enhanced tumor volume. Regarding the dosages and administration of the fluorophores, 5-ALA (20 mg/kg) was administered orally 2.5 to 3.5 h before the induction of anesthesia, and SF (3 mg/kg) was administered intravenously at the induction of anesthesia. In all cases, a Leica M530 OHX microscope (Leica Microsystems, Heerbrugg, Switzerland), equipped with both FL 400 and FL 560 filters to emit and observe different wavelength ranges, was used interchangeably to detect 5-ALA and SF, respectively, and fluorescence pattern distribution.

**Figure 1:**
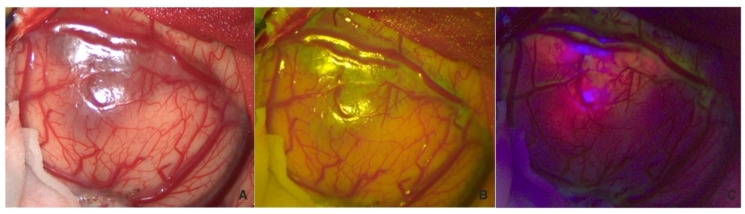
Visualization in white light (**A**), FL 560 (**B**), and FL 400 (**C**) of the tumor before corticectomy.

The surgical strategy involved a first white-light inspection of the tumor, and before excision, the fluorescent pattern was systematically analyzed with both fluorophores to highlight their differences (Figure 1). We can observe an overlap between the two fluorophores (B,C) despite 5-ALA being more prevalent, and structural alteration of the tumor parenchyma is visible even in white light (A).

**Figure 2:**
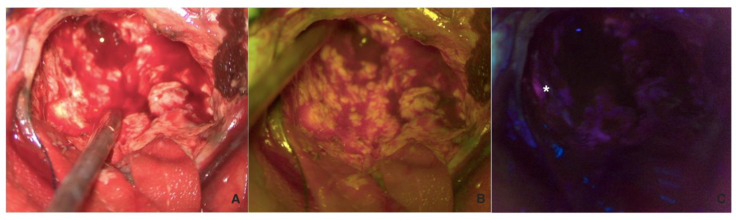
Visualization in white light (**A**), FL 560 (**B**), and FL 400 (**C**) of the tumor during resection. In (**C**), a tumor residual visible on 5-ALA fluorescence, but not on SF or WL, is highlighted with the white asterisk.

Following the corticectomy, the first phase of debulking was performed using only fluorescein (with a shift to white light) to broadly delineate the tumor while easily controlling hemostasis. This phase was followed by a second look using 5-ALA with the possibility of switching between the two fluorophores and white light in cases of doubtful fluorescence, such as in the ependymal region (Figure 2).

**Figure 3:**
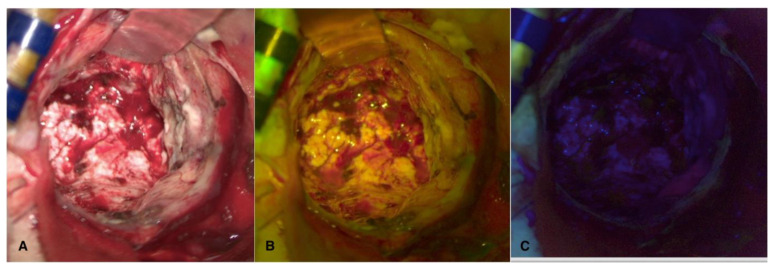
Visualization in white light (**A**), FL 560 (**B**), and FL 400 (**C**) of the cavity after complete resection, with no residual tumor visible with any fluorescent dye.

Finally, the margins of the cavity were explored with both fluorophores in sequence to confirm the absence of fluorescent pathological tissue (Figure 3).

**Figure 4:**
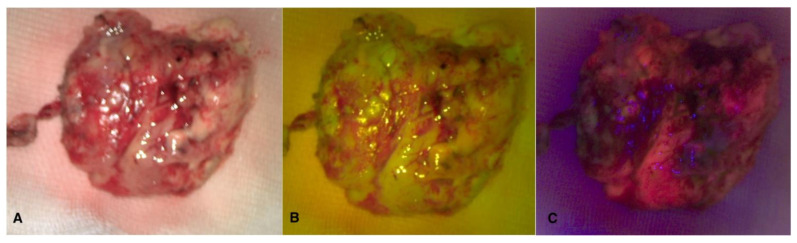
Visualization in white light (**A**), FL 560 (**B**), and FL 400 (**C**) of tumor sample after en-bloc removal.

Intraoperative sample visualization allows us to observe the overlap between the two fluorescences within the tumor nodule (Figure 4).

Resection may be interrupted in cases of alterations in intraoperative neurophysiological monitoring, proximity to white matter bundles highlighted by tractography, or changes in tested functions in cases of awake surgery.

## 3. Case Series

One-hundred patients with histological diagnosis of glioblastoma IDH-Wild Type according to 2021 WHO Classification underwent combined FGR at department of Neurosurgery of AOU City of Health and Science of Turin between 2020 and 2023. Of these, 46 patients were male (46%) and 54 were female (54%). The mean age at the time of surgery was 54.7 ± 13.8 years, and the mean preoperative Karnofsky Performance Status (KPS) was 87.4 ± 12.3. Preoperative neurological deficits were identified in 25 patients (25%). Tumor localization occurred within the frontal lobe in 46 cases (46%), the parietal lobe in 19 cases (19%), the occipital lobe in 9 cases (9%), the temporal lobe in 24 cases (24%), and the cerebellum in 2 cases (2%). Concerning the side, the tumor affected the left hemisphere in 45 cases (45%) and in the right hemisphere in 55 cases (55%). 

The mean time of surgical procedure was 217 ± 79.6 min. Postsurgical complications were identified in seven cases (7%), all of them presenting with intracranial hematomas, and two of them required reoperation (28.5%). The mean postoperative KPS was 84.2 ± 16.8. postoperative neurological evaluation showed an improvement in neurological functions in 13 patients (13%), stability in 53 patients (53%), and worsening in 13 patients (13%). We did not observe any adverse effect possibly related to the administration of the fluorophores (0%)

The extent of resection (EoR) was evaluated according to classification proposed by Karschnia et al. [33], considering the resection of contrast-enhanced (CE) and non-contrast-enhanced (nCE) tissue as follows:

- Class 1 (supramaximal CE resection): 0 cm^3^ CE + ≤5 cm^3^ nCE;
- Class 2 (maximal CE resection):
  ○ Class 2A (complete CE resection): 0 cm^3^ CE + >5 cm^3^ nCE;
  ○ Class 2B (near total CE resection): ≤1 cm^3^ CE;
- Class 3 (submaximal CE resection):
  ○ Class 3A (subtotal CE resection): ≤5 cm^3^ CE;
  ○ Class 3B (partial CE resection): >5 cm^3^ CE;
- Class 4 (biopsy): no reduction in tumor volume.

In our series, supramaximal resection was achieved in 11 patients (11%), complete CE resection in 74 patients (74%), near total CE resection in 9 patients (9%),subtotal CE resection in 3 patients (3%) and partial CE resection in 2 cases (2%). No biopsies were considered for this series. The mean postoperative OS was 18.3 ± 10.7 months. 

All data are summarized in Table 2.

## 4. Discussion

Surgery is currently considered the cornerstone of treatments in GBM patients, particularly due to the importance of the extent of resection as a prognostic value. Recently, Karschnia et al. demonstrated that between different classes of resection (supramaximal CE resection, maximal CE resection, submaximal CE resection and biopsy), there is a statistically significant difference in progression-free survival (PFS) (11 vs. 9 vs. 8 vs. 5 months; *p* = 0.001) and OS (24 vs. 19 vs. 15 vs. 10 months; *p* = 0.001) in GBM patients [33]. In this context, FGR can provide an important support to maximize tumor resection and consequently improve the prognosis of these patients. In fact, this technique provides an intraoperative tool with which to distinguish tumoral tissue from brain parenchyma and guide surgical resection. 

Various studies have evaluated the impact of FGR in the maximization of the EoR and its prognostic value in GBM patients. In a phase 3 randomized trial, the administration of 5-ALA supported by the use of a dedicated BLUE 400 filter showed an increase in the EoR and PFS compared with the control group operated upon under white light. In a study by Stummer et al., complete resection was achieved in 65% of patients in the 5-ALA group vs. 33% of patients in the white light group, which was associated with an improvement in progression-free survival from 20% to 40% [30]. In a recent systematic review, the performance of FGR using 5-ALA has been associated with a greater EoR and with better clinical outcomes compared to white light resection. The authors reported an increase in both PFS (better in 88.4% of cases and no difference in 11.6%) and OS (better in 67.5% of cases and no difference in 32.5%). Moreover, they analyzed postoperative neurological deficits, reporting an improvement in 42.2% of cases, a worsening in 23.3%, and no difference in 34.5% [34].

Up to date, 5-ALA is the only drug approved for FGR, although sodium fluorescein (SF) has seen more and more off-label use by many neurosurgeons in recent years [35]. This trend is related to several advantages of SF over 5-ALA: lower price (EUR 5 per vial for SF vs. EUR 2000 for 5-ALA), easier method of administration in clinical practice (SF is administered intravenously at the beginning of an operation without requiring the darkening of the operating room during surgery), and the possibility of using it in different types of tumors due to its non-specific mechanism of action. In a retrospective cohort study by Hansen et al. including 194 patients diagnosed with new GBM, the authors found no significant difference between 5-ALA and SF use to obtain GTR (64% vs. 62%, *p* = 0.76, respectively), while a better OS was detected in the SF group (14.75 months vs. 19.75 months, *p* = 0.06, respectively). These results suggest that SF could potentially be equivalent and also preferable to 5-ALA in terms of efficacy and outcomes in some cases [36].

Another aspect to take into consideration performing FGR is the risk of false positives. Notably, 5-ALA could present false positivity in both oncological and non-oncological alterations such as peritumoral inflammation, radiation necrosis, and reactive gliosis resulting from chemo-radiotherapies treatments, multiple sclerosis, neurodegenerative diseases, and infectious conditions [37]. Thus, fluorophore uptake must always be considered in relation to the expertise of the surgeon. In a recent meta-analysis, the incidence of false positives was higher in recurrent GBM rather than in newly diagnosed GBM (10.1% vs. 4.1%, *p* = 0.004). Moreover, false positivity in the group of recurrent GBM did not include cases of radionecrosis [38]. Therefore, FGR using 5-ALA is a particularly effective method in newly diagnosed GBM, but also in relapses. Its double fluorescence can help distinguish pathological tumor tissue from scar–glial or post-radiation tissue. This occurs both in cases where the differential diagnosis is with radionecrosis and in cases of recurrent GBM where pseudoprogression is suspected, as the risk of false positives is not negligible and must be considered. Indeed, the role of SF in recurrent GBMs has been explored by Hone et al., achieving GTR in 84% of cases, despite the fact that the distinction of pseudoprogression from recurrent GBM has not been investigated [39]. However, it seems reasonable to assume that SF may have a limited role in discriminating recurrent GBM from pseudoprogression or radionecrosis due to the property of SF distribution according to BBB damage.

Ependymal fluorescence (EF) remains a debated topic in FGR. It is widely described that the opening of the ventricle during FGR using 5-ALA may result in extended fluorescence along the ependymal layer, although the prevalence and the mechanism of this phenomenon are not well understood [40]. According to a recent review, the overall prevalence of EF is around 60%. Nevertheless, the real incidence of EF remains unknown because the ventricle does not need to be opened in all cases [41]. On the contrary, no cases of EF using SF have been reported in the literature [41,42]. This finding supports the combined use of 5-ALA and SF for GBM resection in cases of predicted opening of the ventricle in order to reduce the risk of false positives.

To maximize the advantages of the two compounds and, at the same time, to compensate for their respective limitations, we propose using both 5-ALA and SF in sequential order. Our suggestion is that the first debulking phase is performed under SF guidance, alternating the YELLOW 560 filter with white light to reduce the nonspecific spread of SF in normal brain parenchyma, consequent to the surgical manipulation, and to facilitate the detection of anatomical structures in the early stages of the procedure, thus reducing the risk of accidental damage to vascular or nervous structures [27]. The use of SF as well as white light is indeed less tiring for the surgeon compared to 5-ALA and allows for better management of hemostasis, particularly in lesions that tend to bleed significantly, resulting in a shorter surgical time. On the other hand, we suggest using 5-ALA at a subsequent time, profiting from its higher specificity to individuate tumoral spots within the surgical cavity [35]. Moreover, 5-ALA has also revealed a superiority in terms of sensitivity for infiltrated areas beyond the contrast-enhanced border of the lesion, facilitating supramaximal resection when feasible [43]. Additionally, the delayed use of 5-ALA allows for a reduction in the time spent visualizing under a blue filter, creating a less tiring work environment for surgeons. 

In our series, the subsequential use of 5-ALA and SF allowed us to achieve supramaximal resection in 11/100 cases (11%, Class 1) and complete resection of the contrast-enhanced area in 85/100 patients (85%, Class 1 and 2A), with a positive impact on OS (mean OS: 18.3 months). According to the literature, there is no reported increase in the risk of photosensitive side effects in cases of contemporary administration of both the fluorophores in the same patient; this is probably related to differences in the time of administration, systemic half-life, and pattern of catabolism of the two compounds [44,45]. In our series any side effect that may be linked to the assumption of 5-ALA or SF was observed. Considering economic issues, the low cost of SF in comparison to 5-ALA [17] makes the use of both the dyes economically comparable to the sole use of 5-ALA. Thus, in case of routine use of 5-ALA, the additional use of SF should be considered without provoking a significant modification in the economic burden.

Up to date, only a few studies have evaluated the administration of both 5-ALA and SF [17,18,46,47]. Della Puppa et al. in their series of three cases reported high sensitivity for both 5-ALA and SF (100% and 93%, respectively); the positive predictive value (PPV), negative predictive value (NPV), and specificity of 5-ALA were higher than those of SF (67% vs. 33%, 100% vs. 50% and 94% vs. 87%, respectively). At the same time, both 5-ALA and SF presented a notable false positive rate in which faint fluorescence was present (peritumoral infiltration), although 5-ALA still presented higher specificity than SF (67% vs. 33, respectively) [18]. Zeppa et al. reported an EOR of more than 90% in 87.5% of patients that underwent FGR using 5-ALA, in 77.3% of patients using SF, and in 80% of patients with combined FGR. Regarding OS, combined FGR presented better prognostic outcomes compared with 5-ALA and SF alone, even if statistical significance was not reached for EOR or OS (*p* = 0.783 and *p* = 0.071, respectively) [17].

## 5. Conclusions

Combined fluorescence-guided resection using 5-ALA and SF seems to be a promising method of increasing the extent of resection and improving the prognosis in GBM patients. The combination of the two compounds reduces the limitations associated with the intrinsic characteristics of the fluorophores, without a significant impact on cost or safety compared to the exclusive administration of 5-ALA. We propose the intraoperative subsequential use of the fluorophores to maximize their guidance during surgical resection.

## Figures and Tables

**Table 1:** Fluorescent dyes characteristics. BBB: blood–brain barrier, CE: contrast-enhanced.

	5 Aminolevulinic Acid	Sodium Fluorescein
Wavelength	390–400 nm	460–500 nm
	Metabolic tracer (no need for BBB disruption)	BBB disruption tracer
Timing	4h before anesthesia induction	At anesthesia induction
Photosensitivity	yes	no
Half-life	1–3 h	24 to 36 h
Pitfalls	Ependymal fluorescence, working in low light setting	inferior detection of tumor infiltration beyond CE nodule

**Table 2:** Clinical and surgical features of the sample.

Variables	Value
**Gender**	
Male	46 (46%)
Female	54 (54%)
**Preoperative KPS**	87.4 ± 12.3
**Preoperative neurological deficit**	
Yes	25 (25%)
No	75 (75%)
**Localization**	
Frontal	46 (46%)
Parietal	19 (19%)
Occipital	9 (9%)
Temporal	24 (24%)
Cerebellar	2 (2%)
**Side**	
Left	45 (45%)
Right	55 (55%)
**Age at surgery (years)**	54.7 ± 13.8
**Duration of surgery (minutes)**	217 ± 79.6
**Complications**	
No	93 (93%)
Yes	7 (7%)
Requiring reoperation	2 (28.5%)
**Postoperative KPS**	84.2 ± 16.8
**Postoperative neurological deficit**	
Worsening	25 (25%)
Stability	60 (60%)
Improvement	15 (15%)
**Extent of Resection**	
Class 1	11 (11%)
Class 2A	74 (74%)
Class 2B	9 (9%)
Class 3A	3 (3%)
Class 3B	2 (2%)
Class 4	0 (0%)
**Overall Survival (months)**	18.3 ± 10.7

## Data Availability

The data that support the findings of this study are not openly available due to reasons of sensitivity and are available from the corresponding author upon reasonable request.
